# Temporal Change in Functional Richness and Evenness in the Eastern African Plio-Pleistocene Carnivoran Guild

**DOI:** 10.1371/journal.pone.0057944

**Published:** 2013-03-06

**Authors:** Lars Werdelin, Margaret E. Lewis

**Affiliations:** 1 Department of Palaeozoology, Swedish Museum of Natural History, Stockholm, Sweden; 2 NAMS-Biology, The Richard Stockton College of New Jersey, Galloway, New Jersey, United States of America; University of New South Wales, Australia

## Abstract

We analyze functional richness and functional evenness of the carnivoran guild in eastern Africa from 3.5 Ma to 1.5 Ma, and compare them to the present day. The data consist of characters of the craniodental apparatus of 76 species of fossil and extant carnivorans, divided into four 0.5 Ma time slices from 3.5 to 1.5 Ma, together with the modern fauna. Focus is on large (>21.5 kg) carnivores. Results show that the large carnivore guild has lost nearly 99% of its functional richness since 3.5 Ma, in a process starting prior to 2 Ma. Measurement of functional evenness shows the modern large carnivore guild to be unique in being randomly distributed in morphospace while in all past time slices there is significant clustering of species. The results are analyzed in the light of known changes to climate and environment in eastern Africa. We conclude that climate change is unlikely to explain all of the changes found and suggest that the evolution of early hominins into carnivore niche space, especially the evolution of derived dietary strategies after 2 Ma, played a significant part in the reduction of carnivore functional richness.

## Introduction

As we have demonstrated previously [1], the eastern African carnivoran guild underwent extensive changes in species richness and taxonomic composition during the course of the Plio-Pleistocene. Peak species richness occurred in the early late Pliocene (about 3.6 – 3.0 Ma), with subsequent gradual loss of richness to the present (punctuated by a small peak at 2.1 – 1.8 Ma, most likely due to sampling effects related to the presence of many small carnivoran species at Olduvai, Bed I [2]). Our research has shown that this pattern is the result of a high per capita rate of origination in the late early Pliocene, coupled with a low per capita rate of extinction. In the later Pliocene the origination rate is greatly reduced and the extinction rate somewhat increased, while in the Pleistocene, origination rates are generally low and extinction rates greatly increased.

In a later contribution [3] we discussed the implications for hominin evolution of these changes in richness and turnover. In that context we introduced one ecomorphological variable, body mass, into the analyses, with the specific purpose of limiting the analysis to those species with which hominins would have most likely competed. This includes all species with a body mass of more than ca 21.5 kg. This cutoff point is important from the perspective of carnivoran ecology, since it has been shown [4] that 21.5 kg is a threshold value in carnivoran ecology, with species smaller than 21.5 kg taking mainly small prey (half their own body mass or less) and species larger than 21.5 kg taking large prey (of roughly their own body mass or sometimes greater). We suggested on the basis of these data [3] that hominins of the Plio-Pleistocene must have evolved effective anti-predator strategies in general and effective strategies against kleptoparasitism to compete successfully with the diversity of large-bodied carnivorans present in eastern Africa, particularly during the late Pliocene. Further, we suggested that early Pleistocene hominins, with derived dietary strategies (including increased amounts of animal protein in their diet) and stone tools are implicated in the Pleistocene increase in extinction rate among carnivorans.

These earlier analyses are all taxonomically based and have not taken into account the ecological niches of the taxa involved. Therefore, although we know in a general sense which carnivorans go extinct at what time, the precise ecomorphological pattern of extinctions is not known, nor do we know the effects of these extinctions on the composition and structure of the carnivoran guild through time. This paper addresses these issues, and provides clues as to the effects of the emergence of hominins with derived dietary strategies on the carnivoran guild.

Broad ecomorphological patterns of both fossil and recent carnivorous mammals have been investigated using disparity measures and patterns of morphospace occupation [5]–[7]. We will here build on these studies to analyze patterns of morphospace occupation among eastern African carnivorans from five different time slices from the late Pliocene to Recent. The broad term disparity will here be separated into the more recent concepts of functional richness and evenness [8], [9], and changes to these will form the basis of our characterization of changes through time of the eastern African carnivore guild.

## Materials and Methods

The material analyzed derives from our taxonomic investigations of eastern African fossil carnivorans, as previously outlined by us [1], [3]. The sample used herein consists of a total of 78 species-level taxa, deriving from five 0.5 million year (Ma) time slices: 3.5-3 Ma, 3-2.5 Ma, 2.5-2 Ma, 2-1.5 Ma, and Recent. These samples cover the time from peak carnivoran richness in the early late Pliocene, over the time of the emergence of hominins with derived dietary strategies, to the modern day. After 1.5 Ma the fossil record of eastern African carnivorans is much less well sampled and therefore we have not included time slices within this time interval. Of the 78 species, 29 are of ≥21.5 kg body mass (our body mass categories 3 and 4 [3]), and the remaining 49 are <21.5 kg in body mass. The former group will here be referred to as ‘large carnivorans’ and the latter as ‘small carnivorans’. Only two extant eastern African species have body mass ranges that cross this boundary: the wild dog, *Lycaon pictus* and the cape clawless otter, *Aonyx capensis*. The former is generally larger than 21.5 kg, but some small individuals may be smaller than this, while the latter is generally smaller [10], but some individuals may be larger [11]. For the purposes of this analysis we consider *L. pictus* to be a large carnivoran, but *A. capensis* to be a small carnivoran.

A data matrix including these 78 species, measured for 16 quantitative and semi-quantitative characters of the dental apparatus [5], was constructed. The data matrix is given in Table S1 and a brief description of the characters provided in Appendix S1. Many of the fossil taxa involved are known only from fragmentary material. In the majority of these cases it was possible to reconstruct the full data set for the taxon from several incomplete specimens. When this was not possible, different routes were taken to ensure a complete matrix. Many taxa are conspecific with living species and in such cases data from Recent specimens were used [5], [6], under the assumption that differences within species, even temporally, are substantially smaller than differences between species, either within or between time slices. In cases where the species is extinct, data was reconstructed from congeners. In most of these cases the genera or even families were uniform for the specific character, in which case this character state was used. In a very small number of cases (<10) this was not the case and the character state was reconstructed as the most common state for the genus or species. These net result of these reconstructed states should be to reduce rather than increase morphospace occupation and the analyses therefore err on the conservative side, if at all.

The main measures investigated herein are functional richness and functional evenness [8]. Functional divergence as defined by those authors was not used herein as it requires information on abundance, which was not available to us. The definition of functional richness is ‘the amount of niche space filled by species in the community’ [8]. This was measured by taking the area of the convex hull enclosing all species in the space of the first two axes of a correspondence analysis [12], [13]. We are using only two axes from the 16 available, because all axes after these first two are dominated by the specific features of some taxon or small group of taxa and do not reflect the general patterns we are studying here.

Functional evenness is defined as the evenness of abundance distribution in filled niche space [8]. Since we do not have abundance information available in the fossil data set, we have adapted this definition to our purpose as the evenness of taxon distribution in filled niche space. Although not directly comparable to the measure obtained from the original definition, we suggest that our measure contains information regarding the grouping of taxa in niche space, which has interesting implications for the analysis of developmental pathways and speciation patterns, e.g., with regard to the evolution of hypercarnivory and its constraints [14]–[16].

The distribution of taxa in the space of the first two correspondence axes is presented herein with kernel plots. These provide a direct visual image of taxon distribution, showing hotspots where taxa are closer together in morphospace and gaps where there are no taxa present. Functional evenness is calculated using several alternative methods including the mean and variance in distance between points (taxa), nearest neighbor analysis [17], and Ripley's K [18]. While these two analyses are related, they describe different aspects of evenness. Ripley's K can describe point patterns at a variety of distance scales, such that clustering vs. regularity at both small and large scales can be distinguished (a point pattern may be evenly distributed at small scales, but aggregated at large ones). In nearest neighbor analysis, there is only one scale. Usage of both provides a better understanding of point patterns in the data. The Ripley's K and nearest neighbor analyses are all based on the morphospace of the first two correspondence analyses axes.

Due to the generally small sample sizes, especially of the modern fauna, a number of assumptions are unfortunately violated in the statistical analyses, especially in the case of nearest neighbor analysis, which requires n>50 for adequacy. On the other hand, since the calculations use a Monte Carlo procedure, there is no minimum sample size requirement definable for Ripley's K, and each analysis has to be judged on merit, rather than sampling. In either case, the analyses are suggestive rather than conclusive, but we consider the ensemble of methods, which generally give the same or very similar results for each data set nonetheless to provide a firm foundation for our conclusions.

Statistical calculations were performed using PAST ver. 2.10 [19]. All graphs were made using Aabel ver. 3.0.5 (Gigawiz Ltd.).

Permits to carry out research were obtained from The Office of the President (Kenya), the Tanzania Commission of Science and Technology (COSTECH), and the Authority for Research and Conservation of Cultural Heritage (Ethiopia).

## Results

**Figure 1:**
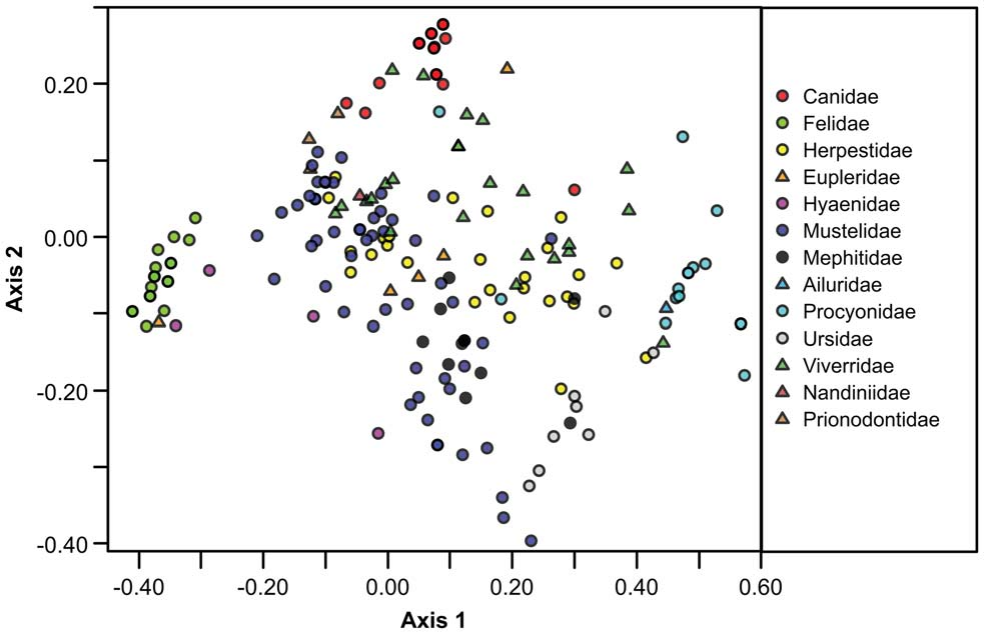
Diagram of the first two axes of the correspondence analysis. The position of each of the families of Carnivora in the morphospace of 216 extant species in our dataset is shown. On Axis 1, note the continuum from hypercarnivores (negative end) to hypocarnivores (positive end). Axis 2 reflects premolar variation, with species with a complete and long premolar row at the positive end and species with reduced or absent premolars at the negative end.

**Figure 2:**
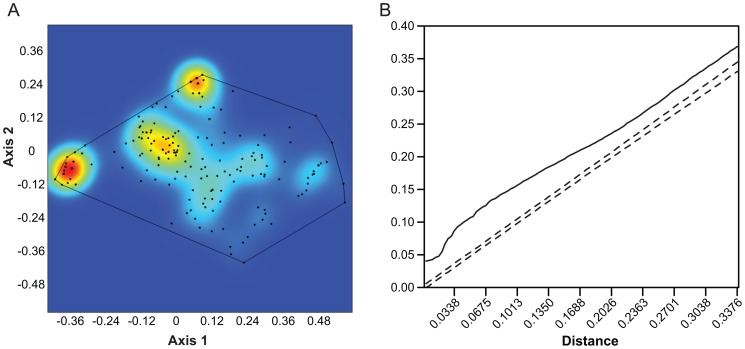
Point pattern analysis of the morphospace occupation pattern seen in **Figure 1**. A. Kernel density plot. Three hotspots at the extreme left (hypercarnivores, mostly Felidae), top (long-snouted, full premolar rows, mostly Canidae), and intermediate (Viverridae, Herpestidae, Mustelidae) are clearly in evidence. B. Ripley's K analysis of the patterns seen in Figure 2A. The x-axis represents inter-point distances, the y-axis the K-value. The dotted lines are the 95% confidence envelope of complete spatial randomness (CSR), while the solid line shows the actual values obtained. Values above the confidence envelope indicate significant attraction between points; values below it indicate significant repulsion between points. In the present case there is significant attraction at all distances.

**Figure 3:**
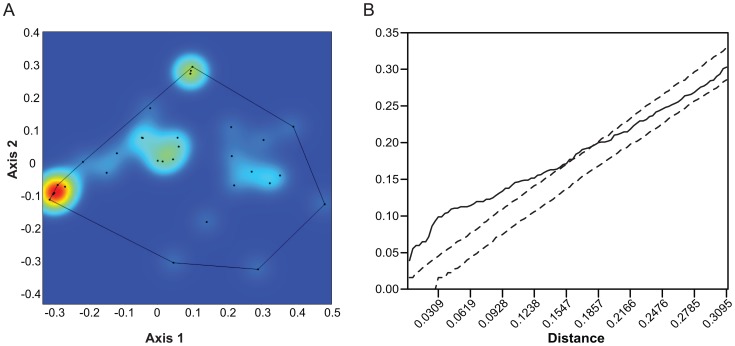
Point pattern analysis of the morphospace occupation pattern of extant African Carnivora. A. Kernel density plot. The same three hotspots as in Figure 2A are seen. B. Ripley's K analysis of the patterns seen in Figure 3A. In this case, there is significant attraction between points at shorter distances (the line lies above the 95% confidence interval limits) but randomness at longer ones (the line lies within the 95% confidence envelope).

**Figure 4:**
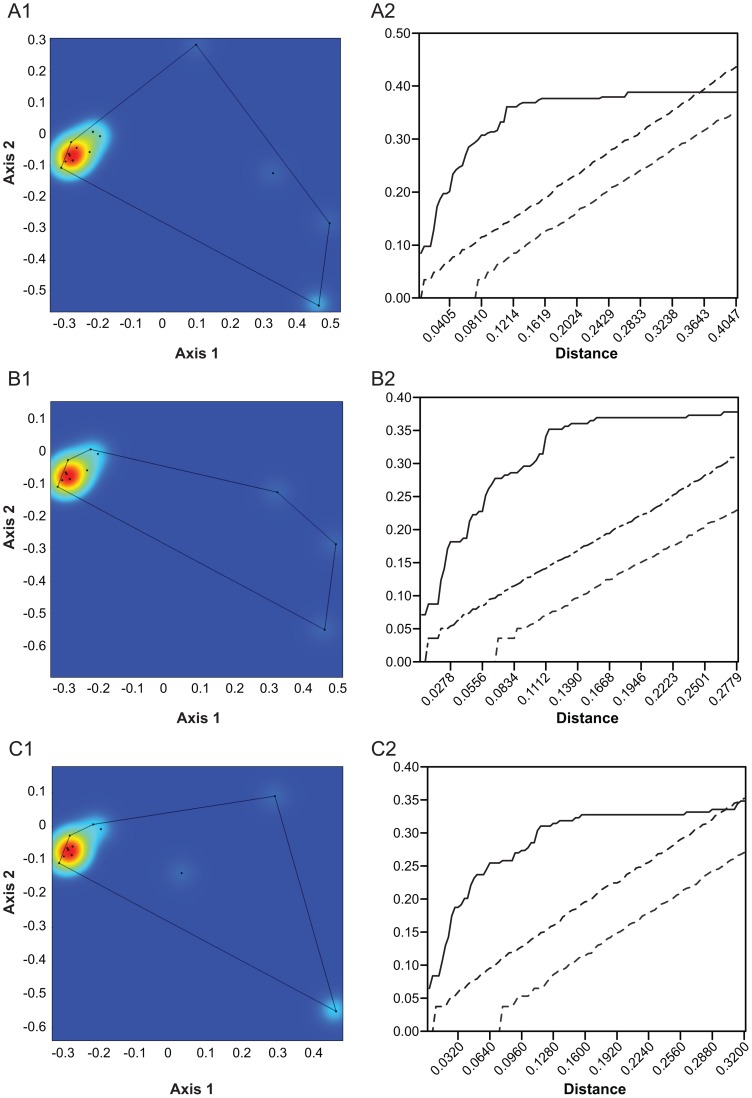
Kernel density and Ripley's K plots of large carnivorans for the first three time slices studied here. A. 3.5 – 3 Ma; B. 3 – 2.5 Ma; C. 2.5 – 2 Ma. Note slight differences in scale between plots. For further discussion, see text.

**Figure 5:**
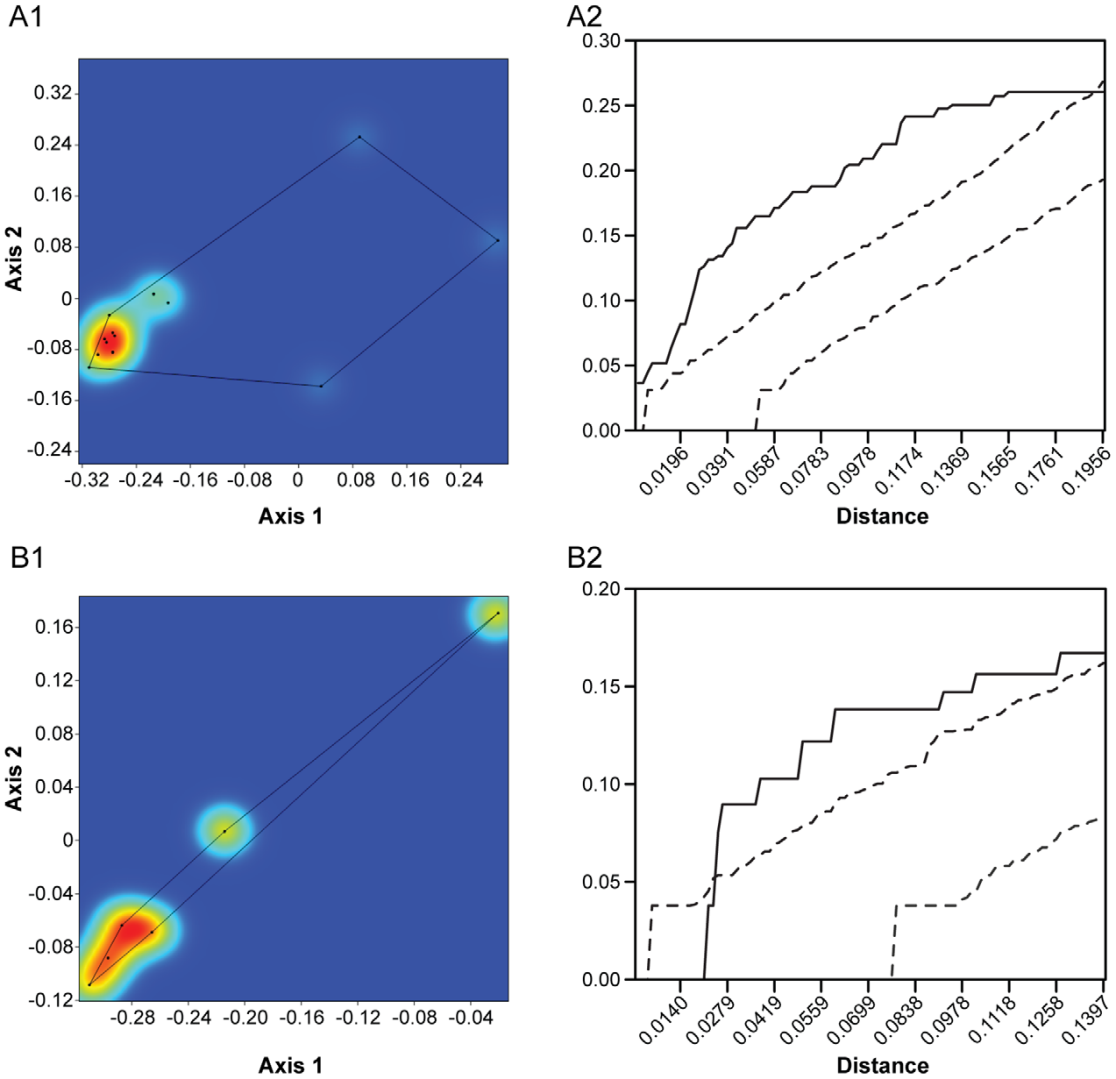
Kernel density and Ripley's K plots of large carnivorans for the final two time slices studied here. A. 2 – 1.5 Ma; B. Extant fauna. Note markedly different scales between these plots and between plots in Figures 4 and 5. For further discussion, see text.

**Figure 6:**
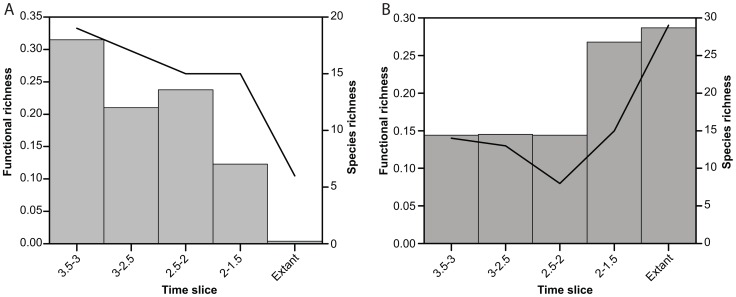
Functional richness and evenness through time. A. Diagram showing changes in functional richness (histogram; left Y scale) and species richness (line; right Y scale) in eastern African large carnivorans through time. B. Same as A, but for small carnivorans. The great difference between A and B is particularly important, see text.

In the following we will first provide some background to the analyses by showing results based on the extant carnivoran guild, globally and in eastern Africa. The data on which this analysis is based are shown in Table S2. These results are essentially those described in Ref. [5], except that all of the present analyses have omitted the body mass variable used in that publication. This section is intended to allow the reader to become visually oriented in the morphospace and are not otherwise an independent analysis. These prefatory graphs are shown in Figures 1 and 2. After this background information is reviewed, we focus on the fossil large carnivorans, presenting results on small carnivorans only when they provide an important perspective on the large ones. The analysis on which these results are based, is graphically represented in Figures 3 and 4. Thus, only two analyses were carried out for the current paper: one of the global extant data set and one of the African extant and fossil data set. The data shown in the Figures 3, 4, 5, and 6 are different extracts from the latter analysis. The scales and units in all analyses are arbitrary but consistent between time slices. It is very important to pay close attention to the very different scales of the axes in Figures 2, 3, and 4.

### Background

Figure 1 shows morphospace occupation in the space of the first two axes of a correspondence analysis of a sample of 216 species from all extant carnivoran families [5]. The first axis can be identified with a continuum from hypercarnivores (negative end) to hypocarnivores (positive end). The most hypercarnivorous extant carnivorans are Felidae, while the most hypocarnivorous are Procyonidae. The second axis is a continuum dominated by premolar variation, with species with a complete and long premolar row (e.g., Canidae) at the positive end and species with reduced or lost premolars (e.g. Lutrini, Ursidae) at the negative end. Overall, families are well discriminated in this analysis, with the exception of Herpestidae and Viverridae [6]. A number of species can be considered outliers relative to their families (see Werdelin and Wesley-Hunt [2010, Fig. 8.2]), but overall the majority of families occupy a relatively restricted area of the total morphospace.

This complete morphospace (n = 216) is represented in the kernel plot shown in Figure 2. The central region clearly has a broad, Y-shaped area of high intensity [20], but there are three hotspots with the very highest intensities. One is the extreme hypercarnivore end of the spectrum, which is chiefly occupied by Felidae, the second is the extreme positive end of the second axis, mainly occupied by Canidae, while the third and largest is a middle ground, but trending more towards the hypercarnivore/long premolar end of the morphospace. This peak is occupied by members of several families, including Viverridae, Herpestidae, and Mustelidae (especially the Mustelini).

Figure 3A shows a similar kernel plot of 35 species of eastern African extant carnivorans. This diagram is not fully comparable to Figure 2 because, as noted above, the two are based on different analyses, but the differences are slight and the interpretation of the axes the same. In eastern Africa too, there are three intensity hotspots (less prominent because there are fewer taxa in this analysis) in the same places as in Figure 2. Thus, if we include all carnivorans, eastern Africa conforms well to the global pattern of morphospace occupation. Statistically, the two differ, however. Ripley's K indicates that the extant global carnivoran guild is significantly aggregated at all distances (Fig. 2B), though more so at shorter distances. The eastern African guild, however, is only aggregated at shorter distances, whereas at longer distances it is randomly distributed (Fig. 3B). Nearest neighbor analysis shows the global pattern to be significantly aggregated, and suggests that the eastern African one is random (Table 1). As nearest neighbor analysis is not multidistance, it is not surprising that it did not detect the aggregation at short distances detected by the K-function.

**Table 1:** Basic statistics of carnivoran morphospace as labeled, and results of the nearest neighbor analyses of these morphospace patterns.

	All extant	all extant EA	large 3.5-3	large 3-2.5	large 2.5-2	large 2-1.5	large extant	small 3.5-3	small 3-2.5	small 2.5-2	small 2-1.5	small extant
N	216	35	19	17	15	15	6	14	13	8	15	29
Area	0.39894	0.29468	0.31507	0.21051	0.23788	0.12351	0.0045823	0.14435	0.14527	0.14435	0.26819	0.28714
Mean density	541.44	118.77	60.303	80.758	63.057	121.44	1309.4	96.989	89.487	55.422	55.931	101
Mean distance	0.016832	0.041606	0.046598	0.031694	0.038984	0.023847	0.026557	0.019307	0.023923	0.053587	0.099	0.042977
Expected distance	0.021488	0.045879	0.064387	0.055639	0.062966	0.04371	0.013818	0.05077	0.052856	0.067163	0.066857	0.04753
Z	−6.092	−1.054	−2.3039	−3.3946	−2.822	−3.5149	4.3205	−4.4359	−3.7757	−1.0938	3.5622	−1.4031
p(random)	1.15E-09	2.92E-01	2.12E-02	6.87E-04	4.77E-03	4.40E-04	1.56E-05	9.17E-06	1.60E-04	2.74E-01	3.68E-04	1.61E-01
R	0.78333	0.90687	0.72372	0.56964	0.61913	0.5256	1.922	0.38029	0.45261	0.079786	1.4808	0.86381

An R-value below one indicates significant attraction between points, an R-value above one indicates significant repulsion between points.

### Descriptive analysis of time slices

In Figure 4A1 we show the kernel plot of large carnivorans of the 3.5 – 3 Ma time slice. This figure, which is derived from the same analysis as Figure 3A and therefore directly comparable, shows a strong aggregation of hypercarnivores. This group is composed of Felidae and Hyaenidae. The peak at the positive end of axis 2 is a single large canid, while toward the lower right of the figure there is a smattering of large omnivores of the families Mustelidae and Ursidae. This time slice is at the time of greatest Plio-Pleistocene species richness of carnivorans [1] and it is not unexpected that it has the greatest functional richness (Fig. 6A, Table 1). As expected from visual inspection of the kernel plot, Ripley's K shows species from this time slice to be significantly aggregated at short distances and only random at very large distances (Fig. 4A2). Nearest neighbor analysis suggests the time slice to be overall significantly aggregated (Table 1).

The 3 – 2.5 Ma time slice shows a similar pattern with essentially the same elements (Fig. 4B1), except that there is no large canid known from the region in that time slice. Instead, the peak along the second axis is lower and occupied by the large-bodied viverrid *Viverra leakeyi*. The lack of a canid makes the functional richness of this time slice lower than that of the previous one (Table 1), but since large canids are known before and after this time slice, this absence may simply be an effect of sampling. Ripley's K shows that species from this time slice are significantly aggregated at all distances (Fig. 4B2), and nearest neighbor analysis indicates the same (Table 1).

The next time slice, 2.5 – 2 Ma is again similar (Fig. 4C1), with a large bodied viverrid, in this case *Pseudocivetta ingens*, providing the peak on the second axis. The Ursidae are no longer present, but large bodied lutrines are, leaving the peak at the hypocarnivore end of the distribution intact. The diagram of Ripley's K is intermediate between the two previous and shows significant aggregation at all distances except the very longest (Fig. 4C2). Nearest neighbor analysis also indicates significant aggregation (Table 1).

In the 2-1.5 Ma time slice, significant changes have occurred (Fig. 5A1). The large bodied hypocarnivores at the lower end of the second axis are now gone and the most hypocarnivorous species is *P. ingens*, while the species that is most negative on axis 2 is an undescribed viverrid from Koobi Fora [21]. These changes mean that functional richness is considerably lower in this time slice than in the older ones, though since species richness has not been reduced, mean species density is considerably higher and mean species distances lower (Table 1). Ripley's K shows significant aggregation at all distances in this time slice (Fig. 5A2). Nearest neighbor analysis conforms to this result (Table 1).

The extant large carnivoran guild shows a completely different pattern (Fig. 5B1). All that remains of functional richness (Table 1) is a small sliver at the hypercarnivore end, with four extreme hypercarnivores (*Panthera leo*, *P. pardus*, *Acinonyx jubatus*, and *Crocuta crocuta*), one intermediate form (*Hyaena hyaena*), and one slightly less hypercarnivorous species (*Lycaon pictus*) at the positive end of axis two. No large bodied hypocarnivores of any kind remain. The validity of Ripley's K (or any other statistical method) is moot here because of the small sample (n = 6), but it does show random distribution at short distances and aggregation at longer ones, which is distinct from previous patterns (Fig. 5B2). Nearest neighbor analysis here indicates significant over-dispersion (Table 1). Regardless of the validity of the statistics, it is clear that the modern guild is very different from the carnivoran guilds at any time in the Plio-Pleistocene.

### Functional richness and evenness through time

Functional richness was measured as the area of the convex hull enclosing all species in the space of the first two axes of the correspondence analysis [12]. The areas are given in Table 1 and shown graphically in Figure 6A (histogram), together with species richness (line). The diagram shows a decline in functional richness of large carnivorans from 3.5 – 3 Ma to 3 – 2.5 Ma, a further decline from 2.5 – 2 Ma to 2 – 1.5 Ma, and a final precipitous decline from 2 – 1.5 Ma to the Recent. This pattern contrasts with that of species richness, which does not decline until well after 2 Ma [1], [3].

A comparison with the pattern shown by the small carnivorans (Fig. 6B) demonstrates that the large carnivoran pattern is not caused by sampling issues. The smaller taxa are affected by known sampling issues, since the majority of finds of carnivorans have been collected from surface surveys and only at a few sites has there been significant sieving done to retrieve microfauna. This means that the representation of small carnivorans (e.g., Herpestidae) is poor except at sites that have been sieved, such as Olduvai. Sieving at that site has increased the representation of small species in the 2-1.5 Ma time slice in comparison to the earlier time slices without sieved sites [1]–[3]. This results in a broader selection of small taxa in that time slice and a concomitantly greater functional richness, despite only a small increase in species richness. Overall, the small carnivorans have low functional richness from 3.5 – 2 Ma, after which it rises to the level of the extant fauna, as one would expect when sampling is an issue. This is completely different from the pattern for large carnivorans and we take this as strong evidence that the large carnivoran pattern is not driven by sampling vagaries.

**Figure 7:**
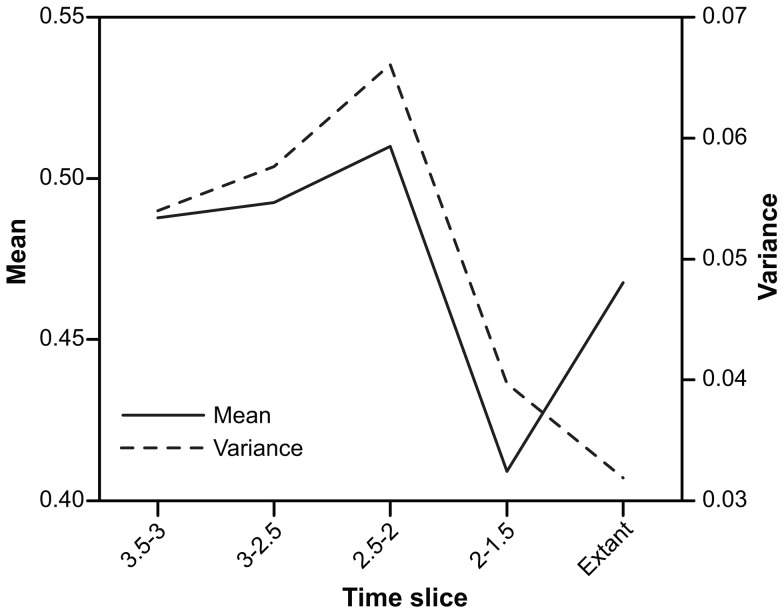
Inter-point mean and variance through time. Diagram showing changes in the mean (solid line) and variance (dotted line) inter-point distance in carnivoran morphospace through time.

Functional evenness is a more difficult concept to measure and evaluate. However, the point pattern analyses (Ripley's K, Nearest neighbor) suggest that the past time slices are similar in that all show significant, strong aggregation of species in morphospace, while the extant guild shows a random or nearly random distribution in morphospace, at least at short inter-point distances. A third way of measuring functional evenness is as the variance of the mean distance between points in morphospace. This mean and variance are shown in Figure 7. The mean can be viewed as a measure of disparity [22], while as stated above, the variance measures functional evenness, i.e., the distribution of species in morphospace.

In the three oldest time slices, the mean is stable (increasing only slightly over time), after which it drops dramatically in the 2 – 1.5 Ma time slice as a result of the decrease in functional richness alongside stable species richness (leading to more species per unit area and shorter inter-point distances). In the extant fauna, the mean (disparity) has again increased, because although functional richness is very low, species richness has also decreased.

The pattern of functional evenness (variance) is similar to that of disparity until the extant fauna, in which variance has decreased to a very low level, despite disparity having increased. Hence, in the extant carnivoran guild, species are more evenly distributed in morphospace than at any time in the past, leading to a unique situation of low functional richness accompanied by low functional evenness.

## Discussion

Several problems arise when attempting to address the causal basis of the patterns described above. The first of these is the lack of precision of the analysis. Given the data available at the present time, time slices smaller than 0.5 Ma reduce sample sizes to the point where vagaries of sampling lead to random fluctuations in functional richness. This is because the presence or absence of a single taxon has (or can have) greater influence on these patterns than on patterns of species richness and turnover, where time slices as small as 0.3 Ma were used [1]. For similar reasons we expect changes in taxonomic richness to lag behind changes in functional richness. This means that we expect changes in the latter to predate changes in the former, but by an unknown amount of time. Thus, our previous results [1], [3], which clearly showed that the major loss of species richness among eastern African carnivorans occurred after 2 Ma, are compatible with those of the present paper, which show that major changes in functional richness began after 2.5 Ma but before 2 Ma. Together, these two results show that, whatever the cause of the decline in carnivoran richness, it was a process that extended over hundreds of thousands of years, and was not a single, temporally restricted “event.”

A second problem is that the critical time interval is one in which several interconnected processes took place that may have affected carnivoran richness, independently or together. These include climatic change (begun earlier but continuing during this time interval), biome change (causally linked to climatic change, but in a non-linear fashion), and changes within the human lineage that caused greater overlap and competition with the carnivoran guild. We must also bear in mind that there are other components of the larger carnivore guild beyond carnivorans and hominins (e.g., avian and crocodylian members) whose effects are not taken into account in this analysis.

Given these problems, we can only some preliminary answers to the question of causality. This we will do by combining previous work by many authors on the processes mentioned above with the specific nature of the relationship between large carnivorans on the one hand and the surrounding abiotic and biotic environment on the other hand. We will also compare the situation with what is known of large carnivore richness patterns on other continents in the present day.

The pattern of climate change in the Plio-Pleistocene of eastern Africa is by now well established [23], [24]. A long-term trend towards climatic cooling was punctuated by stepped increases in climate variability and aridity around 2.8, 1.7, and 1.0 Ma [24]. These changes resulted in an opening-up of the eastern African landscape, where generally forested habitats were replaced by more open grasslands [25]. The result was a complex mosaic of habitats as these over-arching trends were mediated by local conditions of temperature and precipitation. The three step-episodes have been of particular interest in the study of faunal change because of their connection to the turnover-pulse hypothesis of Vrba [26], [27], which suggests that turnover in mammalian lineages occurred in episodic fashion mediated by pulses of climatic change. In the context of the present paper, the 1.7 and 1.0 Ma episodes are too late in time to provide a causal explanation of the primary pattern of change beginning prior to 2 Ma, but the 2.8 Ma episode is clearly of interest and we will focus on that episode in the following discussion.

Although the turnover-pulse hypothesis as originally proposed has not found support in the majority of subsequent studies, there is a general consensus that the time interval 2.8 – 1.8 Ma is a time of increased turnover in the eastern African fauna, albeit with considerable regional and taxonomic variation [28]–[30]. Importantly, there is an increase in turnover among Bovidae, Suidae, Cercopithecidae, and Hominidae in this interval, peaking around 1.9 Ma [29]. The same authors also show a gradual increase in the number of grassland indicators among mammalian taxa in this interval and beyond. Finally, they show a peak in faunal change early in the interval, followed by lower change and then increased variability. Regardless of this internal variation in the analyses, the consensus is clear and the connection to the climatic episode around 2.8 Ma is circumstantial but convincing, at least as far as these taxa, all primary consumers, are concerned.

Carnivorans, however, show a somewhat different pattern. We have previously shown [1], [3] that the time period 3 – 2.1 Ma is a time of minimum turnover among carnivorans, and that increased turnover, leading to a decline in species richness, did not begin in earnest until after 2 Ma and peaked after 1.8 Ma. This difference can be interpreted in two ways – either the carnivorans were affected by the same climatic and environmental changes as the primary consumers but with a lag time required for the changes to trickle up to the secondary and tertiary consumers [31], or the effects on carnivorans of the climatic and environmental changes in the half million years after 2.8 Ma were small (or at least not detectable at the scale of analysis). However, referring the carnivoran turnover pattern to climatic changes beginning at 2.8 Ma creates the problem of what amount of lag time to accept and still consider two phenomena to be due to the same primary cause. In the present case, we would have to accept an extensive lag time to allow changes in species richness among primary consumers to affect the secondary and tertiary consumers. There is no theoretical and little empirical evidence for the length of such a lag time, but what little evidence there is from analogous situations such as Late Pleistocene megafauna extinctions in the Americas (regardless of cause) [32]–[35] suggests that the lag time, if any, should be measured in thousands or tens of thousands of years, rather than hundreds of thousands.

We conclude from this that the climatic changes initiated at the 2.8 Ma episode and their subsequent environmental effects did not directly lead to the loss of species richness observed in carnivorans after 2 Ma. However, we have in this paper shown that functional richness in carnivorans began to decrease earlier than species richness, so significant guild changes related to the 2.8 Ma episode may be masked by a robustness in species richness among carnivores. How this may happen can be seen from Figures 3 and 4, in which the ‘hotspots’ of functional richness can be seen to lie in the extreme hypercarnivory and extreme ‘long snout’ ends of the distribution, with a third hotspot between them. The hypocarnivorous and short-faced ends of the distribution show much lower densities. Thus, regardless of whether extinctions specifically target those low density areas of the morphospace or are random with regard to morphospace position, loss of functional richness can be expected to occur mainly in those areas, with hotspots remaining in the hypercarnivore and long snout regions, which is partly what we see. The hypercarnivore hotspot persists throughout all time slices and in the fossil time slices is composed of 10, 9, 9, and 10 species respectively, from 3.5 – 1.5 Ma (Fig. 4). The other hotspots seen in Figures 2 and 3 are, however, not in evidence in Figure 4. They are composed mainly of smaller taxa of several families and are of lesser significance among large carnivores. Nevertheless, apart from a loss of the “long snout” area in the 3 – 2.5 Ma time slice (Fig. 4B1), this part of morphospace remains constantly occupied, even to the present day, unlike the more hypocarnivorous parts of morphospace, which are lost from at least 2 Ma onward.

Hence, that the loss of functional richness was not random, but was at first (prior to 2 Ma) concentrated on hypocarnivores and those with reduced premolar rows (generally large-sized omnivores). Not until after 2 Ma, and probably nearer 1.5 Ma were hypercarnivores and long-faced omnivores (Canidae) affected. This makes a connection for hypercarnivores to the 2.8 Ma climatic episode untenable, while for hypocarnivores and the short-faced omnivores this connection remains plausible but must be considered on a case-by-case basis and in conjunction with other potential causal factors.

One such potential causal factor is the appearance of derived hominins (the genus *Homo*) sometime after the 2.8 Ma climatic pulse. A characteristic of the evolution of *Homo* over the subsequent million years is the likely increase in the proportion of animal proteins in the diet, leading to an introgression of *Homo* into carnivore niche space [36]. This effectively made *Homo* a part of the carnivoran guild, and would have led to increased levels of competition with carnivorans. Many authors have discussed this process from the hominin perspective [37]–[41], but fewer have considered the effect of hominins on the carnivoran guild [3], [42].

It is by now well established that the earliest *Homo* (*H. habilis*, *H. rudolfensis*) had not yet evolved the derived dietary strategies seen more derived hominins [43]. However, at least some features had evolved that indicate a broadening of the dietary spectrum. Foremost among these is the development of stone tools, first recorded at ca 2.6 Ma [44]. These two developments in hominin evolution must have impacted on the carnivoran guild in various ways. The impact of hominins with derived dietary strategies is in clear evidence, as we have noted previously [3]. However, functional richness shows changes prior to 2 Ma and may be more sensitive to the more subtle changes wrought by the first steps of hominins into carnivore niche space.

The pattern seen in hominins takes them step-wise from a more herbivorous diet to a diet incorporating more proteins of animal origin. On this basis we predict that the effects on the carnivoran guild should first be seen among omnivores and, if some arguments are valid, among exploiters of aquatic food resources [45]. Not until later, in the post-2.0 Ma step, would they have had a major effect on hypercarnivores. This is the pattern that we see. Omnivore/piscivore/molluscivore morphospace has become significantly reduced in the 2–1.5 Ma time slice as a result of a loss of functional richness prior to this time, while hypercarnivore morphospace remains undisturbed. Not until after 1.5 Ma are hypercarnivores affected, chiefly through the extinction of sabertooth predators along with some hyenas, such as *Chasmaporthetes* prior to 1.5 Ma.

Of course, it is also possible to build an equally valid scenario around a climatic cause for the reduction of omnivore morphospace, via a cooling and drying climate to a loss or reduction in large, permanent bodies of water and forested environments ideal for omnivores and piscivores. Without further analysis it is not possible to say which causal factor is the driving force behind the changes in carnivoran functional richness prior to 2 Ma, or if both play a part and if so, which is the more important. A direct role for climate in the later changes to the hypercarnivore part of carnivoran morphospace requires a far more elaborate set of indirect consequences stemming from changes to the primary consumer communities, which leaves more room for competitive interactions, especially with hominins.

## Conclusions

The analyses presented here are necessarily preliminary and much work is needed to determine the causal chain that resulted in the depauperate carnivoran functional richness seen today. Some avenues of continued research that potentially can tease apart these factors can nevertheless be suggested. One is a comparison between eastern and southern African patterns. This has been hampered to date by the poor temporal control on South African Plio-Pleistocene faunas. The situation in this regard is rapidly improving [46]–[48], and soon an analysis such as the one presented here can be carried out on South African material as well. A second research direction is to look at other continents. For example, studies by Palombo and colleagues [49]–[51] suggest that an increase in carnivoran extinctions in Europe occurred around 0.5 Ma, the time of the first long-term hominin settlements in on that continent outside the Iberian Peninsula. A competitive role for humans in the much later extinction of carnivorans (and other megafauna) in North America at the end of the Pleistocene has also been suggested [35]. A third research direction is for an increase in research on the non-mammalian carnivores of Africa to provide a more holistic understanding of the greater carnivore guild to which carnivorans (and eventually hominins) belong.

## Supporting Information

Appendix S1 **Descriptions of the characters used in the analyses.**
(DOCX)

Table S1 **Taxon coding used in the African extant/fossil analyses.** Legend for Time slice column: 1: 3.5-3 Ma; 2: 3-2.5 Ma; 3: 2.5-2 Ma; 4: 2-1.5 Ma; 5: extant.
(XLSX)

Table S2 **Taxon coding of 216 extant carnivoran taxa.**
(XLSX)
