# Supplementary material for: Temporal Change in Functional Richness and Evenness in the Eastern African Plio-Pleistocene Carnivoran Guild
Source: PLoS One. 2013 Mar 6;8(3):e57944. doi: 10.1371/journal.pone.0057944 (PMC3590191; doi:10.1371/journal.pone.0057944)
Supplement: Appendix S1 — Descriptions of the characters used in the analyses. (DOCX) [file pone.0057944.s001.docx]

**Supplementary information, Appendix.**

Character Descriptions. Further discussion and illustrations of characters is provided elsewhere [[1](#_ENREF_1),[2](#_ENREF_2)].

1. *Incisor row: parabolic or straight*. (1) parabolic organization, (2) straight incisor row.

2. *Canine: length over width*, measured at the enamel-dentine junction. This is an ordered, continuous character. The continuum was divided into five categories based on the distribution and the extreme morphology of saber-toothed forms: (1) X ≤ 1.2, (2) 1.2 < X ≤ 1.35, (3) 1.35 < X ≤ 1.5, (4) 1.5 < X ≤ 1.7, (5) X > 1.7.

3. *Number of upper premolars anterior to the carnassial*. Vestigial premolars are not included. This character is discrete and ordered.

4. *Largest upper premolar anterior to the carnassial: length over width*. This is an ordered, continuous character. The continuum was divided into three categories based on its distribution. The three bins are defined as: (1) X ≤1.7, (2) 1.7 < X ≤ 2.3, (3) X > 2.3.

5. *Upper premolar spacing: close or spaced*. Premolars were characterized as spaced if gaps were present between the first, second, and third upper premolar. Premolars were characterized as close under the following conditions: 1) no space between the second and third premolar, however there may be space present between the canine and the first premolar, or between the first and second premolar, but not both; or 2) the first and second premolar are vestigial.

6. *Last lower premolar: length over width*. This character is ordered, and continuous. The continuum was divided into three categories based on its distribution, the exact cut-offs between categories were arbitrary: (1) X < 1.7, (2) 1.7 ≤ X ≥2.2, (3) X > 2.2.

7. *Shape of the upper carnassial*: (1) square, (2) equilateral triangle, (3) an elongate triangle (approximating a right, scalene triangle), (4) linear. The shape is determined by the outline of the occlusal surface. The carnassial is classified as linear if the protocone participates in the shear and there is no shelf lingual to the protocone.

8. *Blade length of upper carnassial*: length of shearing blade compared to total length of the upper carnassial. (1) no blade present, (2) the blade 1/3 of total length, (3) the blade 1/2 of total length, and (4) the blade 2/3 or greater of total tooth length. This character is ordered.

9. *Relative blade length of lower carnassial*: ratio of the anteroposterior length of the trigonid, measured on the buccal side, over the total maximum length of the tooth. This character is ordered and continuous in distribution. The distribution is divided into five categories: (1) X = 0 (2) 0 < X < 0.55 (3) 0.55 ≤ X ≤ 0.75, (4) 0.75 ≤ X ≤ 0.9, (5) X > 0.9.

10. *Angle β, upper carnassial*. ß is the angle between a line drawn from the metacone to the most anterior projection of the parastyle, and a line drawn from the metacone to the apex of the protocone, with the tooth positioned in full occlusal view. This character is ordered. The continuum was divided into four categories based on the distribution, and distinguishing the extremes: (1) X < 24°, (2) 25° ≤ X < 30°, (3) 30° ≤ X < 40°, (4) X ≥ 40°.

11. *Angle α, lower carnassial*. α is the angle between a line drawn along the base of the tooth crown, above the roots, and a line drawn tangential to the protoconid and to the highest point on the talonid with the tooth positioned in full occlusal view. This character is ordered. The continuum was divided into five categories based on the distribution: (1) 0°< X < 15°, (2) 15° ≤ X < 30° (3) 30° ≤ X < 50° (4) 50° ≤ X < 70° (5) X ≤ 70°.

12. *Angle γ lower carnassial*. γ is the angle between the paralophid and protolophid of the trigonid of the lower carnassial positioned in occlusal view. This character is ordered. The continuum was divided into 5 categories: (1) X = 0°, (2) 0° < X ≤ 40°, (3) 40° < X ≤ 80°, (4) 80° < X ≤ 130°, (5) X = 180°. If the metaconid is absent there is no protolophid, and the angle is coded as 180° (category 5) which is characteristic of hypercarnivorous forms.

13. *Shape of upper first molar*: 1) square or longitudinal rectangle, 2) transverse rectangle, 3) triangle, 4) absent. Shape was determined by outline of the occlusal surface.

14. *Number of upper molars*. For a molar to be considered present, its occlusal surface area must be equal to at least one half of the surface area of the first upper molar. This character is ordered.

15. *Cusp shape*. Cusps on the upper first molar were classified as round or sharp. “Sharp” is defined as a cusp that comes to a point, and possesses sides with straight slopes. A “round” cusp has a rounded tip and the sides have curved slopes.

16. *Grinding area of the lower molars*. Calculated as the total occlusal surface area of the lower molars divided by the total grinding surface area: surface area of m1+m2+m3…)/ (m1 talonid+m2+m3+grinding area of p4. Modified from Relative Grinding Area. This character is ordered, and continuous. The continuum was divided into five categories beginning with the entire molar region dedicated to grinding and ending with no grinding area at all: (1) X=1, (2) 1 < X < 2.25, (3) 2.25 ≤ X ≤ 4, (4) 4 < X ≤ 6 (5) X > 6. The last bin represents all taxa with a grinding area of 0 or a grinding ratio greater than 6.

**References**

1. Werdelin L, Wesley-Hunt GD (2010) The biogeography of carnivore ecomorphology. In: Goswami A, Friscia A, editors. Carnivoran Evolution: New Views on Phylogeny, Form, and Function. Cambridge: Cambridge University Press. pp. 225-245.

2. Wesley-Hunt GD (2005) The morphological diversification of carnivores in North America. Paleobiology 31: 35-55.
